# Atmospheric Pollution Exposure Increases Disease Activity of Systemic Lupus Erythematosus

**DOI:** 10.3390/ijerph17061984

**Published:** 2020-03-18

**Authors:** Paula Henriques Blaskievicz, Ageo Mario Candido Silva, Vander Fernandes, Osvaldo Borges Pinto Junior, Walkiria Shimoya-Bittencourt, Silvana Margarida Benevides Ferreira, Cristhiane Almeida Leite da Silva

**Affiliations:** Master’s Program in Environment and Health, University of Cuiabá, Cuiabá 78065-700, Brazil; bridahenriques@hotmail.com (P.H.B.); ageoms@hotmail.com (A.M.C.S.); vf@uol.com.br (V.F.); osvaldo.borges@kroton.com.br (O.B.P.J.); wshimoya@yahoo.com.br (W.S.-B.); silvana_benevides@hotmail.com (S.M.B.F.)

**Keywords:** systemic lupus erythematosus, air pollution, disease activity

## Abstract

Previous studies have shown that high levels of air pollutants may increase activity of systemic lupus erythematosus (SLE). The aim of this study is to analyze the association between pollutants originating from the Brazilian Legal Amazon and SLE activity. This is a retrospective longitudinal cohort study with patients with SLE in the General Hospital in Cuiabá, Brazil. The association with SLE activity was measured using the SLE disease activity index (SLEDAI) and data on air quality—PM_2.5_ and CO, published on the websites of the State Department of Environment and the Center for Weather Forecasting and Climate Studies. To assess the effect of daily concentrations of pollutants on SLEDAI scores, the generalized estimation equation (GEE) model was used. A total of 32 female patients were assessed, in 96 doctor’s appointments. The average SLEDAI score was 6 points (±5.05). GEE showed an association of disease activity with both higher rates of wildfires (*p* = 0.021) and average CO rate (*p* = 0.013), but there was no statistical association between particulate levels and SLE activity. The results suggest that variations in air pollution are associated with the activity of autoimmune rheumatic diseases.

## 1. Introduction

Systemic lupus erythematosus (SLE) is a chronic, autoimmune, and multifactorial inflammatory disease characterized by great complexity and broad clinical spectrum, with periods of exacerbation and remission [1,2,3,4]. The disease is characterized by loss of immune tolerance to various autoantigens and formation of immune complexes that deposit in the tissues and induce inflammation in different organs and systems [5].

Although there is a clear genetic predisposition to many autoimmune diseases, environmental factors play a key role in determining whether the genetic factor can manifest itself [2,6,7]. Previous research has focused on different environmental factors, and it has been found that inhalation of air pollution may affect susceptibility to autoimmune diseases and triggers the development of a systemic inflammatory reaction [8].

Recent data have suggested that these types of exposure may be important triggers for systemic inflammation in SLE. Fernandes et al. [9], in a study carried out in the city of São Paulo, Brazil, found an increase in the disease activity of juvenile systemic lupus erythematosus, associated with increased concentration of particulate matter 12–15 days before manifestation of disease activity. Exposure to concentrations of fine particulate matter, which are sometimes considered to be unhealthy, may lead to increased levels of some serum inflammatory markers. In addition to SLE, these markers are indicative of other pathologies [9,10,11]. For example, Makar et al. [11], while evaluating air pollutants and respiratory diseases, found that increased long-term exposure to PM_2.5_ from levels below 8 µ/m^3^ to levels above 8 µ/m^3^ led to a 15% increase in the hospitalization rate for such diseases. 

According to Zeft et al. [12], Vidotto et al. [4], and Fernandes [9], few studies to date have evaluated the association between exposure to air pollution and onset or exacerbation of autoimmune diseases. Zeft et al. [12] found a higher risk of onset of symptoms of juvenile idiopathic arthritis in children under five years, associated with higher PM concentrations_2.5_ and stagnant air conditions in the last 14 days (RR = 1.60, 95%, CI 1.00–2.54). 

Other pollutants have been evaluated for rheumatic diseases. Vidotto et al. [4], in a time-series study from January 2000 to December 2007, evaluated the influence of air pollution exposure on the daily number of hospitalizations caused by exacerbation of seven pediatric rheumatic diseases, including systemic lupus erythematosus. The authors found, for a variation of an interquartile range of SO_2,_ a 1.98% increase (CI = 0.25–3.69) in the number of hospitalizations caused by acute exacerbations of the study diseases after 14 days of exposure. Thus, knowledge of environmental factors and their mechanisms in autoimmune diseases are a relevant issue because such diseases are chronic and disabling.

The generation of air pollutants has increased exponentially, especially in urban centers; thus, air pollution has become one of the major health risk factors. In the last decades, the literature has collected a very solid series of epidemiological evidence and associated air pollution with harmful health effects and increased mortality [13,14,15]. According to Kampa and Castanas [16], these harmful effects occur even under usual concentrations of pollutants in various urban centers. 

Biomass burning is one of the world’s leading contributors to air pollutant emissions, including greenhouse gases and particulate matter, which often results in human exposure to high levels of air pollutants. Although forest fires and deliberate biomass burning are intermittent sources of air pollution, they represent major sources of combustion pollution on a global scale [17].

Therefore, the aim of this study was to evaluate the association of variations in atmospheric pollutant concentrations on systemic lupus erythematosus activity. There is a lack of research in this field, especially in Brazil and in the city of Cuiabá, in Brazil’s Amazon Region, where air pollution resulting from biomass burning is common.

## 2. Material and Methods

The present study is a retrospective longitudinal study using a repeated measures design, conducted between June 2010 and February 2014, at the General University Hospital (HGU/UNIC), located in Cuiabá, capital of the state, in the Central-South mesoregion of Mato Grosso. The city currently extends over an area of 3,291,816 km², with an estimated population of 533,801 inhabitants [18]. It is situated in the Brazilian Legal Amazon, which is considered to be the gateway to the Amazon forest [19].

The study population consisted of female patients diagnosed with systemic lupus erythematosus who had already been receiving treatment for SLE and were monitored between 06/2010 and 02/2014. The patients lived in Cuiabá, were aged 18 years or older, used the Rheumatology Outpatient Service of the University General Hospital and were diagnosed with systemic lupus erythematosus (ICD 10 M32), according to criteria of the American College of Rheumatology (ACR) [20]. They were followed up during three consecutive clinical evaluations. Patients were excluded from the study if they did not live in Cuiabá and if their records about disease activity were incomplete. 

This study was approved by the Research Ethics Committee of the University General Hospital, University of Cuiaba, under registration No. 081/UNIC, Protocol 2010-062. All the participants gave their informed consent.

Disease activity was recorded in each appointment using the systemic lupus erythematosus disease activity index (SELENA-SLEDAI). Data on carbon monoxide and particulate matter were collected within 14 days prior to each doctor’s appointment by air pollutant dispersion modeling obtained from the coupled aerosol and trace gases transport model to the Brazilian developments on the Regional Atmospheric Modeling System (CATT-BRAMS), available on the institutional website of the National Institute for Space Research (INPE) [21]. Information about air temperature, relative humidity and radiation index was provided by the Center for Weather Forecasting and Climate Studies of the National Institute for Space Research (CPTEC/INPE) [22].

Continuous variables were presented as mean ± standard deviation or median and variation, according to the variable distribution (Komogorov–Smirnov test), and categorical variables were presented as absolute and relative values. Explanatory variables fire occurrence on the 14 days preceding the appointments (yes/no); mean values for PM_2.5_, CO, and temperature; and the medians of relative humidity of the last 14 days prior to the doctor’s appointment. Comparison of the means was performed by Student’s *t*-test; and the correlations between PM_2.5_ and CO were determined by Pearson’s correlation coefficient and scatter plots. The association between the effect of daily pollutant concentrations on SELENA-SLEDAI scores was analyzed using the generalized estimating equation (GEE) model, while considering fixed effects for repeated measures. This study has a longitudinal design and includes repeated measurements on the same subject, i.e., each participant is observed at different follow-up times. Therefore, neither repeated measures variance analysis nor multiple linear regression should be used because neither of them meets the assumption of independence of random variables, given the interdependence relationship of measurements on the same subject [23]. GEE analysis is based on the generalized linear models methodology, initially proposed by Liang and Zeger [24] for the context of longitudinal data; it is able to generate regression estimates more accurately when these data are autocorrelated [25]. A *p*-value ≤ 5% was considered as statistically significant.

## 3. Results

During the study period (June 2010 to February 2014), 69 patients were evaluated in 96 appointments, 15 in the second appointment and 12 in the third appointment. The patients’ mean age at the first appointement was 39 years (±10.49), with a minimum of 20 years and maximum of 62 years. The most common skin color was brown (53.1%), followed by black (34.3%), and white (12.5%). The patients had been living with SLE for an average period of 9.7 years, at the date of the first appointment. Patients’ mean age at the time of diagnosis of SLE was 26.7 years. 

The assessment of the scores achieved through SLEDAI in the three appointments showed that the mean score ranged from 6.63 points (±4.61) in the first appointment to 5.97 points (±4.80) in the third appointment (Table 1).

Table 2 shows the data on the pollutants analyzed in the three appointments. Mean concentration of carbon monoxide ranged from 0.06 ppm (±0.07) in the first appointment to more than double in the third appointment (0.13 ppm ± 0.07). PM_2.5_ concentration ranged, on average, from 7.38 µm (±6.92) in the first appointment to 14.12µm (±5.04), indicating an increase in the average level of these pollutants over the years.

Regarding climatic variables, temperature ranged from 27.83 °C (±1.89) in the second appointment to 28.45 °C (±2.47) in the third appointment, and relative humidity ranged from 54.93%. (±13.85) to 56.98 (±11.19) in the third appointment. Data on the insolation variable ranged from 5.87 Gy (±1.61) the first appointment to 8.75 Gy (±13.75) in the second appointment. 

Table 3 shows the means of CO and PM_2.5_ concentration according to the presence/absence of disease manifestation in the three appointments. CO was significantly associated with myocarditis, myositis, and osteonecrosis (*p* < 0.005). Regarding PM_2.5_, all manifestations evaluated were statistically associated (*p* < 0.05).

**Figure 1:**
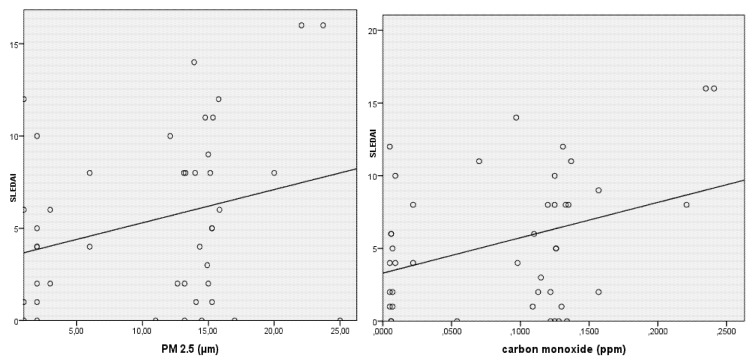
Correlation between PM_2.5_ and CO with variation in the systemic lupus erythematosus disease activity index (SLEDAI) score (Pearson’s correlation coefficient).

In the generalized estimating equation (GEE) model, with variation in the SLEDAI score as the dependent variable and the other climate and air pollution variables as explanatory variables, the following variables remained associated with increased SLEDAI score: carbon monoxide (*p* = 0.013), fires (*p* = 0.021), and the fire-carbon monoxide interaction (*p* = 0.001). The PM_2.5_ variable was not statistically associated with SLEDAI variation (Figure 1) and (Table 4).

Figure 1 shows the Pearson’s correlation coefficient between CO and PM_2.5_ concentration and SLEDAI scores in the three appointments. Positives correlations were statistically associated in both correlation tests.

## 4. Discussion

The aim of the present study was to evaluate the association between some atmospheric pollutants originating from biomass burning and anthropogenic sources in the Amazonian biome on the exacerbation of systemic lupus erythematosus activity. The main variable associated with increased SLEDAI was carbon monoxide. Few studies to date have evaluated the effects of air pollutants inhaled by patients with rheumatic diseases. Bernatsky et al. [26] evaluated the association of PM_2.5_ and the SLEDAI-2K score in 237 lupus patients monitored from 2000 to 2007, and they found that total SLEDAI-2K scores were not associated with increased levels of PM_2.5_. However, their findings suggest that short-term variations in particulate matter concentration may trigger exacerbation of systemic lupus erythematosus. 

The adverse effects of air pollution on human health have been recognized for decades, and while it is well understood how air pollution affects the lungs, its effects on other systems are not as evident [6]. The study of Seaton et al. [27] was widely known for proposing the hypothesis that the systemic effects of air pollution exposure are mediated through the induction of pulmonary oxidative stress and inflammation, whose mediators are carried by the bloodstream and generate effects at a distance.

However, several studies have also associated the influence of biomass burning on other diseases, in addition to autoimmune diseases. Quinn et al. [28] found an association between exposure to carbon monoxide from wood stoves and increased blood pressure in pregnant women. In another study conducted in Palermo, Italy, Tramuto et al. [10] found a positive association between hospitalizations for respiratory problems and carbon monoxide from vehicle pollution.

Some studies with controlled exposure of patients using fine particulate matter have shown changes in inflammation-related biomarkers [14,29]. With regard to the immune system, air pollution has attracted attention from researchers as a factor that may influence allergic disease; however, involvement with other types of hypersensitivity reactions has been poorly studied [6,8,27,30]. Autoimmune diseases are a group of disorders that can affect a wide variety of organs and systems, with one common feature: the immune system is inappropriately activated and produces destructive responses against its own antigens [6].

Although some studies have not focused directly on the SLEDAI outcome, in 2009, Calderón-Garcidueñas et al. [31] found that children exposed to high levels of PM_2.5_ developed systemic inflammation, thus showing that there was a significant correlation between higher concentrations of PM_2.5_ and increased levels of CRP and PGE2. These results confirm previous findings that exposure to environmental pollution can lead to the development of a systemic inflammatory process.

Air pollution is a heterogeneous mixture that includes gases, liquids, and solid components, and each one of them has unique potential effects on biological systems. In general terms, the adverse effects of air pollution are believed to occur through induction of the NF-κB pathways and MAP kinase in response to oxidative stress [6]. Volatile compounds and reactive metals can directly generate free radicals. In the case of air pollution, oxidative stress can still be produced by inert particles [32]. These effects are not located in the respiratory microenvironment, but they have systemic effects: systemic oxidative stress, bone marrow stimulation, increase in the number of leukocytes and cytokine levels in the blood, and maturation of antigen-presenting cells, thereby providing the immune system with costimulatory molecules expressed by T lymphocytes [6,32].

It is speculated that air pollution may influence autoimmunity by facilitating autoantigen presentation in a context that could cause activation of autoreactive T lymphocytes [6]. Air pollution generates oxidative stress in the airways, causing epithelial cells and alveolar macrophages to express proinflammatory cytokines that lead to dendritic cell maturation, migrate to lymph nodes and present autoantigens to autoreactive T lymphocytes, thereby triggering autoimmune responses. Once the autoimmune response is established, even in cases in which air pollution was not the triggering factor, proinflammatory cytokines generated by exposure to air pollution will consolidate and exacerbate responses [6].

In Mato Grosso, burning of sugarcane forests and crops is the major source of air pollution, and these emissions have been responsible for causing diseases in people of different age groups [33,34]. 

In addition to biomass burning exposure as a source of CO, this phenomenon also occurs in large urban centers (with the exception of smokers, who are directly contaminated by CO), where motor vehicles, together with factories, are the main sources of air pollutants. People who spend several hours a day in vehicles or who are directly exposed to smoke emissions are the most affected. However, indoor environments such as homes and offices can also suffer the effects of CO from outside air pollution through ventilation systems, or CO produced on-site by oil heaters, barbecue grills, gas stoves or even by smokers [35].

Carbon monoxide has 240 times as much affinity for hemoglobin as oxygen [36,37], which causes a small amount of CO to saturate a large amount of hemoglobin molecules, decreasing the blood’s ability to carry O_2_. It also shifts the hemoglobin dissociation curve to the left, leading to a decreased release of O_2_ in the tissues [38].

Particulate matter, considered as one of the main solid components of burnt smoke, was not associated with the outcome when included in the GEE model. Nevertheless, it is known that the health of the population affected by smoke from fires is impacted by the components present in the heterogeneous mixture of gases and particles. Even when pollutant concentrations of this mixture are below the air quality standards established by legislation in Brazil and in other countries, this exposure triggers the increase of various types of diseases in the population [11,39,40].

In this sense, in the city of São Paulo, Fernandes et al. [39] found an increase in juvenile systemic lupus erythematosus associated with an increase in the concentration of particulate matter between 12 to 15 days prior to the doctor’s appointment for disease evaluation. Exposure to concentrations of fine particulate matter, sometimes considered as unhealthy, may raise the level of some serum inflammatory markers, which indicate the manifestation of pathologies other than SLE. [11,13] Makar et al. [11], when evaluating air pollutants and respiratory diseases, found that increased long-term exposure to PM_2.5_ from levels below 8µ/m^3^ to levels above 8µ/m^3^ led to a 15% increase in the hospitalization rate for these diseases.

Recent studies have associated air pollution with an increase in chronic inflammatory diseases, including autoimmune diseases [8]. Zeft et al. [15] found a higher risk of onset of juvenile idiopathic arthritis symptoms in children under five years old associated with higher PM_2.5_ concentrations and stagnant air conditions over the last 14 days (RR = 1.60, 95%, CI 1.00–2.54).

Vidotto et al.^4^ in a time-series study, evaluated the influence of air pollution exposure on daily number of hospitalizations caused by exacerbation of seven pediatric rheumatic diseases, including systemic lupus erythematosus, from January 2000 to December 2007. For a variation of an interquartile range of SO_2,_ the authors found a 1.98% increase (CI = 0.25–3.69) in the number of hospitalizations because of acute exacerbations of the study diseases after 14 days of exposure.

Importantly, the determination of the US Environmental Protection Agency (EPA) to control particles smaller than or equal to 2.5 µm, also called inhalable particles, was based on the fact that these are particles can reach the lower airways. Such inhalable particulate matter may carry adsorbed gases on its surface to the most distal portions of the airways where gas exchange occurs in the lungs [13]. Particulate matter less than 10 µm in diameter (PM_10_) can penetrate the respiratory tract; however, it is stopped on the way to the bronchioles. By contrast, particulate material smaller than 2.5 µm is absorbed by the alveoli, reaching the bloodstream. The action of these pollutants decreases mucociliary and macrophage activity, producing airway irritation; it also causes oxidative stress and, as a consequence, pulmonary and systemic inflammation [41].

In the present analysis, using the GEE model, a first-order interaction was found between CO and the concomitant occurrence of fires. This result suggests that, in addition to the direct effect of air pollution, the combined presence of these two variables leads to an even greater exacerbation of SLE in the study population. Strong evidence shows that air pollution exposure is detrimental to the health of the population, but in the Amazon Region, pollution is seasonal and occurs in high concentrations, as seen in fires and in drier periods of the year [42].

For the meteorological variables, no statistically significant associations were found between the increase of SLEDAI and the variables “average temperature, relative humidity and insolation.” One of the possible explanations is that the model considered the average values of these variables on the 14 days prior to the doctor’s appointment; thus, these values were possibly “smoothed.” In addition, these data are collected in fixed stations; therefore, they do not fully reflect the variation in individual exposure, as people are likely to spend most of their time indoors or at work.

Environmentalists, scientists, and society at large criticize the use of fires in natural fields and forests, but this type of resource is common in tropical and subtropical regions, especially those with a very dry season. In the Brazilian Amazon, this is a common situation; small fires and large forest fires near pasture fields are often started by farmers. This is also the case of the state of Mato Grosso, where this habit clearly causes several problems to the population’s health [43]. A survey of the National Institute for Space Research [44] pointed out that there were 9070 fire outbreaks between August the 16th and 17th, 2010. As a result, more than three million tons of carbon monoxide were released into the atmosphere in the state of Mato Grosso alone, from the beginning of the year until mid-August 2010.

The city of Cuiabá is located in the middle of the savannah of Mato Grosso, a part of the Legal Amazon. In this region, a part of the air pollutants come from anthropogenic sources (mobile and stationary) and another part comes from pasture burning, forest fires, and fires in backyards. Intense fires in southern Amazon occur in an adjacent region near Cuiabá. However, as a preventive measure, there is a prohibitive period of fires that starts in July and ends in October. During this period, government agencies monitor the occurrence of hotspots in an attempt to prevent the most critical situations of drought and intense atmospheric pollution, which are common at that time of the year [45].

Estimation of pollution concentration by the CATT-BRAMS model [44] is performed by a computer system designed to simulate and study the atmospheric transport of products originating with biomass burning, thereby estimating the concentration of PM_2.5_ and CO in the Amazon region and in Cuiabá. According to Silva et al. [46], the estimates of this model were compared with actual on-site measurements of PM_2.5_, and they were validated and found to be satisfactory. However, one limitation of this study is the use of secondary data on the pollutants. In addition, daily information on PM_2.5_ and CO had some missing data; therefore, information on SLEDAI scores was not included when such scores were collected from appointments scheduled on those days. Another limitation was the pharmacological effects of the drugs prescribed to the study patients, because they may change the SLEDAI score. These effects are difficult to interpret by the statistical method used in this research; thus, they were not evaluated in the present data analysis.

This study found that exposure to air pollution can be a major trigger for SLE disease activity. Government policy-making, as well as measures to improve education and public awareness, are crucial to reduce air pollution, in order to bring about an improvement in the quality of life of the population.

## 5. Conclusions

Our data suggest that exposure to carbon monoxide and pollutants originating from biomass burning increases disease activity in SLE patients. 

## Figures and Tables

**Table 1:** Descriptive data of number of appointments for each patient and the systemic lupus erythematosus disease activity index (SELENA-SLEDAI) variable in each appointment.

SLEDAI	*N*	%	Mean	Median	SD	Variance	Min.	Max.
1^st^ appointment	69	71.9	6.63	5.00	4.61	21.27	0.00	16.00
2^nd^ appointment	15	15.6	6.16	5.50	5.07	25.75	0.00	17.00
3^rd^ appointment	12	12.5	5.97	4.00	4.80	23.03	0.00	16.00

**Table 2:** Descriptive data on the pollutants in the three appointments.

	Mean	Median	Standard Deviation	Variance	Minimum	Maximum
CO (PPM)						
1st appointment	0.06	0.01	0.07	0.01	0.01	0.25
2^nd^ appointment	0.08	0.10	0.06	0.00	0.01	0.24
3^rd^ appointment	0.13	0.13	0.07	0.00	0.01	0.25
PM_2.5_ (µm)						
1st appointment	7.38	2.50	6.92	47.83	1.00	22.00
2^nd^ appointment	10.93	13.92	7.46	55.70	1.00	25.00
3^rd^ appointment	14.12	14.68	5.04	25.40	1.00	23.71
Temperature (°C)						
1st appointment	28.38	28.50	1.56	2.44	23.90	30.80
2^nd^ appointment	27.83	28.50	1.89	3.58	23.00	30.80
3^rd^ appointment	28.45	27.80	2.47	6.12	23.60	32.70
Humidity (%)						
1st appointment	55.58	59.00	15.26	232.86	24.00	81.46
2^nd^ appointment	56.98	56.35	11.19	125.28	27.71	71.00
3^rd^ appointment	54.93	55.00	13.85	191.71	26.50	82.92
Insolation (Gy)						
1st appointment	5.87	5.70	1.61	2.58	1.90	8.60
2^nd^ appointment	8.75	6.80	13.75	189.806	3.70	82.5
3^rd^ appointment	6.32	6.90	1.96	3.84	2.50	9.60

**Table 3:** Means and standard deviation of CO and PM_2.5_ concentration according to the presence/absence of disease manifestation.

Disease Manifestation	CO (PPM)				PM_2.5_ (µm)			
	**Yes**		**No**			**Yes**	**No**	
	**Mean**	**SD**	**Mean**	**SD**	**Mean**	**SD**	**Mean**	**SD**
Interstitial lung disease	0.131	0.054	0.091	0.071	15.081	3.9411	11.177	6.805 *
Myocarditis	0.163	0.079	0.085	0.061 *	17.716	5.114	10.654	6.280 *
Myositis	0.175	0.087	0.083	0.056 *	18.498	7.455	10.514	5.685 *
Osteonecrosis	0.206	0.049	0.087	0.062 *	19.603	2.629	10.978	6.378 *
Pericarditis	0.132	0.068	0.093	0.069	15.787	4.705	11.177	6.653 *
Pleuritis	0.130	0.070	0.089	0.070	15.621	4.302	11.088	6.714 *

* *p* < 0.05.

**Table 4:** Data on air pollutants and SLEDAI (generalized estimating equation (GEE)).

Dependent Variable: Variation in SLEDAI Score				
Parameters	β1	Standard Deviation	Chi-square	Significance
Fires	1.464	0.636	5.296	0.021
CO	8.451	3.410	6.141	0.013
PM_2.5_	0.012	0.034	0.119	0.730
Fires+CO	10.800	3.198	11.401	0.001
